# Culture and Next-generation sequencing-based drug susceptibility testing unveil high levels of drug-resistant-TB in Djibouti: results from the first national survey

**DOI:** 10.1038/s41598-017-17705-3

**Published:** 2017-12-15

**Authors:** Elisa Tagliani, Mohamed Osman Hassan, Yacine Waberi, Maria Rosaria De Filippo, Dennis Falzon, Anna Dean, Matteo Zignol, Philip Supply, Mohamed Ali Abdoulkader, Hawa Hassangue, Daniela Maria Cirillo

**Affiliations:** 1 Emerging Bacterial Pathogens Unit, Division of Immunology, Transplantation and Infectious Diseases, IRCCS San Raffaele Scientific Institute, Milan, Italy; 2 Hopital pneumo-phtisiologie Chakib Saad, Djibouti, Djibouti; 3 National TB Reference Laboratory, Djibouti, Djibouti; 4 World Health Organization/Global TB Programme, Geneva, Switzerland; 5 CNRS Institut Pasteur de Lille, Lille, France; 6 Programme National de Lutte contre la Tuberculose, Djibouti, Djibouti

## Abstract

Djibouti is a small country in the Horn of Africa with a high TB incidence (378/100,000 in 2015). Multidrug-resistant TB (MDR-TB) and resistance to second-line agents have been previously identified in the country but the extent of the problem has yet to be quantified. A national survey was conducted to estimate the proportion of MDR-TB among a representative sample of TB patients. Sputum was tested using XpertMTB/RIF and samples positive for MTB and resistant to rifampicin underwent first line phenotypic susceptibility testing. The TB supranational reference laboratory in Milan, Italy, undertook external quality assurance, genotypic testing based on whole genome and targeted-deep sequencing and phylogenetic studies. 301 new and 66 previously treated TB cases were enrolled. MDR-TB was detected in 34 patients: 4.7% of new and 31% of previously treated cases. Resistance to pyrazinamide, aminoglycosides and capreomycin was detected in 68%, 18% and 29% of MDR-TB strains respectively, while resistance to fluoroquinolones was not detected. Cluster analysis identified transmission of MDR-TB as a critical factor fostering drug resistance in the country. Levels of MDR-TB in Djibouti are among the highest on the African continent. High prevalence of resistance to pyrazinamide and second-line injectable agents have important implications for treatment regimens.

## Introduction

Tuberculosis (TB) strains with multidrug-resistance (MDR-TB; resistance to rifampicin and isoniazid) and extensive drug-resistance (XDR-TB; MDR-TB with additional resistance to any fluoroquinolone plus one or more of amikacin, kanamycin or capreomycin) represent a major threat for global TB control. Mortality and other unfavorable outcomes are higher among MDR-TB patients on treatment^1^. Access to diagnosis is still problematic in many countries and treatment is long and more costly than that for drug-susceptible TB. In most resource-limited countries drug-susceptibility testing is not routinely performed on all newly diagnosed TB patients mainly because of the lack of laboratory capacity and infrastructure. In recent years, molecular technologies have been swiftly replacing conventional phenotypic testing thus simplifying laboratory workload and logistics, improving diagnostic access and reducing the cost for testing^2^. Sequencing, and particularly whole genome sequencing (WGS), has been recently evaluated in industrialized countries^3^ for drug resistance surveillance and it is under evaluation in resource limited settings. In addition to drug resistance prediction, whole genome sequencing allows to differentiate clinical isolates into the major *Mycobacterium tuberculosis* (*Mtb*) lineages and sub-lineages which is particularly relevant in the context of national drug resistance surveys given that specific lineages are intrinsically resistant to certain anti-TB agents^4^, or seemingly acquire mutations at a higher rate^5^ and have been associated with MDR-TB development^6^.

Djibouti, a small country located in the Horn of Africa with less than 1 million inhabitants, has a high TB incidence (378 cases per 100,000 population in 2015) and TB mortality (43 per 100,000 population)^1^. In recent years, the national TB programme (NTP) of the Ministry of Health has introduced WHO-endorsed molecular diagnostics (XpertMTB/RIF and GenoType MTBDR*plus* assay) to enhance the local capacity to detect and treat TB patients^7^. Nonetheless, treatment success has been suboptimal largely as a result of interruptions (81% in new and relapse cases in 2014 cohort, with 12% of patients lost to follow-up)^1^. The emergence of drug-resistant TB strains represents a major concern for the NTP. In 2015, 85 cases of MDR-TB as well as 7 cases of XDR-TB were officially reported^1^. However, precise estimates of the prevalence of MDR-TB among new and previously treated TB cases in Djibouti are lacking as the country does not have a continuous drug resistance surveillance system in place and no national drug resistance survey has ever been conducted.

National surveys conducted in neighboring countries have shown various levels of MDR-TB, ranging from 2.7% (95%CI 1.5–4.0) and 14% (95%CI 5.6–23) among new and previously treated TB cases respectively in Ethiopia in 2005, to 2.3% (95%CI 0.92–3.7) and 18% (95%CI 11–25) among new and previously treated TB cases respectively in Yemen in 2011^1^, and up to 5.2% (95%CI 2.8–7.5) and 40.8% (95%CI 24.7–57.0) among new and previously treated TB cases detected in Somalia in 2011^8^.

In this paper, we describe the first national TB drug resistance survey conducted by the NTP of Djibouti to estimate the burden of MDR-TB in the country and to provide information about additional resistance to other TB drugs (pyrazinamide, fluoroquinolones and injectable agents). These data help the NTP and its supporting partners with resource prioritization as well as planning and implementation of effective prevention and curative measures.

## Results

During the study period, a total of 367 pulmonary TB patients, of which 301 were new and 66 previously treated, were included in the survey. Table 1 summarizes the demographic and clinical data of patients stratified according to treatment history. Among new and previously treated cases, 70% were male, the median age was 30 years for both male and female (interquartile range [IQR]: 23–43). More than 90% of the enrolled cases were residing in the city of Djibouti and 23.2% were of foreign origin, mainly from Ethiopia (17.7%). Information on comorbidities was available for 100% of patients. The prevalence of HIV among the enrolled population was 7.1% (26 HIV positive cases) and that of diabetes 6.3% (23 cases).

**Table 1:** Distribution of socio-demographic and clinical characteristics of enrolled patients, by treatment history, Djibouti nationwide drug-resistance survey, 2014–2015.

		New cases (n = 301)		Previously treated cases (n = 66)		Total (n = 367)	
		n	%	n	%	n	%
Sex	Male	209	69.44%	47	71.21%	256	69.75%
	Female	92	30.56%	19	28.79%	111	30.25%
Age	0–14	9	2.99%	2	3.03%	11	3.00%
	15–24	79	26.25%	10	15.15%	89	24.25%
	25–34	103	34.22%	18	27.27%	121	32.97%
	35–54	78	25.91%	28	42.42%	106	28.88%
	55–64	13	4.32%	4	6.06%	17	4.63%
	≥65	19	6.31%	4	6.06%	23	6.27%
Country of origin	Djibouti	230	76.41%	52	78.79%	282	76.84%
	Eritrea	1	0.33%	0	0%	1	0.27%
	Ethiopia	56	18.60%	9	13.64%	65	17.71%
	Senegal	1	0.33%	0	0%	1	0.27%
	Somalia	13	4.32%	5	7.58%	18	4.90%
Setting	rural	19	6.31%	5	7.57%	24	6.54%
	urban	282	93.69%	61	92.42%	343	93.46%
Smoking	no	163	54.15%	35	53.03%	198	53.95%
	yes	138	45.85%	31	46.97%	169	46.05%
Alcohol use	no	301	100%	65	98.48%	366	99.73%
	yes	0	0%	1	1.52%	1	0.27%
HIV	negative	283	94.02%	58	87.88%	341	92.92%
	positive	18	5.98%	8	12.12%	26	7.08%
Diabetes	no	284	94.35%	60	90.91%	344	93.73%
	yes	17	5.65%	6	9.09%	23	6.27%

DRS = drug resistance survey.

### Prevalence of Rifampicin resistance and MDR-TB

Rifampicin resistance was detected by XpertMTB/RIF testing in 36 patients representing 9.8% (95%CI 7.2–13.3%) of TB cases, with a prevalence of 4.3% (95%CI 2.5–7.3%) among new and 34.9% (95%CI 24.5–46.9%) among previously treated. Resistance was confirmed by phenotypic drug susceptibility at the National reference laboratory on 32 (88.9%) rifampicin-resistant cases and results were 100% concordant with genotypic testing. For the remaining 4 rifampicin-resistant cases, phenotypic confirmation was not possible due to culture contamination.

Either whole genome sequencing or targeted sequencing was performed on 35/36 (97.2%) rifampicin-resistant and 191/331 (57.7%) rifampicin-susceptible sputum samples. Targeted sequencing allowed to identify an additional case of rifampicin resistance due to a Ser450Leu mutation that was originally missed by XpertMTB/RIF due to heteroresistance. Rifampicin resistance was thus confirmed in 37 patients representing 4.7% (95%CI 2.8–7.7%) of newly diagnosed (n = 14) and 34.9% (95%CI 24.5–46.9%) of previously treated TB cases (n = 23) (Table 2).

**Table 2:** Prevalence of resistance to Rifampicin and MDR-TB in smear-positive patients with pulmonary TB, Djibouti nationwide drug-resistance survey, 2014–2015.

	New cases (n = 301)		Previously treated cases (n = 66)		Total (n = 367)	
	n	% (95%CI)	n	% (95%CI)	n	% (95%CI)
Rifampicin-resistance	14	4.7% (2.8; 7.7)	23*	34.9% (24.5; 46. 9)	37	10.1% (7.4; 13.6)
MDR-TB	14	4.7% (2.8; 7.7)	20	30.8% (20. 9; 42.8)	34	9.3% (7.2; 14.6)

^*^Isoniazid susceptibility testing was available for 22 out of 23 rifampicin resistant previously treated TB patients.

All the 14 newly diagnosed rifampicin-resistant TB cases were also resistant to isoniazid, meaning that the prevalence of MDR-TB was also 4.7% (95%CI 2.8–7.7). Among the previously treated group, isoniazid resistance was detected in 20 out of 22 (90.1%) rifampicin-resistant cases tested, resulting in a prevalence of MDR-TB of 30.8% (95%CI 20.9–42.8) (Table 2).

### Pattern of resistance to pyrazinamide and second line drugs in MDR-TB cases

Table 3 shows the resistance profile to pyrazinamide and second line drugs among MDR-TB cases detected by genotypic testing. Pyrazinamide (PZA) resistance-conferring mutations in *pncA* gene and its promoter region were detected in 23 isolates representing 67.7% (95%CI 50.8–80.9%) of MDR-TB resistant strains. Resistance to second-line injectable agents was detected by genotypic drug susceptibility testing in 6 isolates representing 17.7% (95%CI 8.4–33.5) of MDR-TB cases all carrying the resistance-conferring mutation 1401 g in the *rrs* gene. In addition, 4 isolates carried a specific mutation (Asn236Lys) in the *tlyA* gene^9^ and were phenotypically resistant to capreomycin leading to an overall prevalence of capreomycin resistance of 29.4% (95%CI 16.8–46.2%). Conversely, none of the MDR-TB nor rifampicin resistant isolates carried mutations leading to fluoroquinolone resistance.

**Table 3:** Proportion of resistance to pyrazinamide and second-line anti-TB drugs among patients with MDR-TB, Djibouti nationwide drug-resistance survey, 2014–2015.

		New cases (n = 14)		Previously treated cases (n = 20)		Total (n = 34)	
Drug	Resistance profile	n	% (CI95%)	n	% (CI95%)	n	% (CI95%)
PZA	R	10	71.43 (45.4; 88.3)	13	65.0 (43.3; 81.9)	23	67.7 (50.8–80.9)
AMK/KAN	R	2	14.3 (4.0; 39.9)	4	20.0 (8.1; 41.6)	6	17.7 (8.4; 33.5)
CAPREO	R	3	21.4 (7.6; 47.6)	7	35.0 (18.1; 56.7)	10	29.4 (16.8; 46.2)
FQs	R	0	0 (0.0; 21.5)	0	0 (0.0; 16.1)	0	0 (0.0; 10.2)

PZA: pyrazinamide; AMK: amikacin; KAN: kanamycin; CAPREO: capreomycin; FQs: fluoroquinolones; R: resistant.

### Pattern of resistance in rifampicin-susceptible samples

A total of 191 (57.9%) rifampicin susceptible samples underwent genotypic drug susceptibility testing by either whole genome or targeted next generation sequencing. The remaining 139 (42.1%) rifampicin susceptible samples were not available for genotypic analysis because specimens had already been used for re-testing in country (e.g. first XpertMTB/RIF invalid or error) or only one sample was referred to the national reference laboratory. Isoniazid resistance conferring mutations were detected in 9 (4.7%) TB cases, 8 new and 1 previously treated. Similarly, a mutation in the *pncA* gene was detected in only one (0.5%) rifampicin-susceptible sample but its role in drug resistance development is unknown^10,11^. Thirteen (6.8%) rifampicin susceptible isolates belonged to the *M. canetti* lineage which is known to be intrinsically resistant to pyrazinamide^4^. Whole genome sequencing analysis revealed the presence of a specific pyrazinamide resistance conferring mutation (Met117 Thr) in the *panD* gene^12^ in 100% of the *M. canettii* cases tested.

While none of the rifampicin-susceptible samples carried mutations responsible for fluoroquinolone, amikacin or kanamycin resistance, 6 (3.1%) of them carried the Asn236Lys mutation in *tlyA* gene and were phenotypically resistant to capreomycin.

### Factors associated with multidrugresistance

Table 4 shows the results of univariate and multivariate analysis. In multivariate analysis, MDR-TB prevalence was significantly higher in patients previously exposed to anti-TB drugs (odd ratio [OR] 11.6; 95%CI 4.9–27.0; p < 0.0001). The odds of MDR-TB were also higher among individuals with diabetes (OR 9.9; 95%CI 3.1–31.5; p < 0.0001), but not among HIV-positive patients (OR 0.8; 95%CI 0.2–3.6). In addition, individuals ≥ 55 years were more likely to have MDR-TB (OR 3.7; 95%CI 1.3–10.4; p = 0.001) as opposed to the younger group.

**Table 4:** Analysis of factors associated to MDR-TB development, Djibouti nationwide drug-resistance survey, 2014–2015.

Univariate logistic regression			Multivariate logistic regression		
	OR (95%CI)	p-value		OR (95%CI)	p-value
**History of TB treatment**			**History of TB treatment**		
New cases	1		New cases	1	
Previously treated	9.1 (4.2; 19.3)	<0.0001	Previously treated	11.6 (4.9; 27.0)	<0.0001
**Diabetes**			**Diabetes**		
no	1		no	1	
yes	8.8 (3.4; 22.7)	<0.0001	yes	9.9 (3.1; 31.5)	<0.0001
Age group*			Age ≥55*		
0–14	1		no	1	
15–24	0.9 (0.1; 7.7)	0.89	yes	3.7 (1.3;10.4)	<0.0001
25–34	0.6 (0.1; 5.5)	0.66			
35–54	0.9 (0.1; 8.2)	0.95			
55–64	3.1 (0.3; 32.0)	0.35			
≥65	3.5 (0.4; 33.7)	0.27			
**Sex**					
Female	1				
Male	0.8 (0.4; 1.6)	0.51			
**HIV**					
Negative	1				
Positive	0.8 (0.2; 3.6)	0.77			
**Setting**					
rural	1				
urban	1.1 (0.3; 5.1)	0.87			
**Foreign origin**					
No	1				
Yes	0.5 (0.2; 1.5)	0.22			
**Smoking**					
No	1				
Yes	1.4 (0.7; 2.8)	0.39			

*Univariate analysis for Age ≥55 shows OR of 4.2 (95%CI 1.8–9.6); p = 0.001.

### Phylogenetic profile of MTB complex strains

Table 5 shows the lineage classification of MTB strains isolated during the survey. The lineages of 207 (56.4%) samples (91.9% of rifampicin resistant and 52.4% of rifampicin susceptible samples) out of the 227 analyzed by either whole genome or targeted next generation sequencing could be identified and classified into the main phylogenetic lineages according to Coll^13^. The lineage of 20 samples analyzed by targeted sequencing could not be univocally identified and the samples were thus excluded from the analysis. More than 90% of the MTB isolates belonged to the three major lineages 4, 1 and 3 (48.3%, 20.8% and 22.7% respectively), while 6.3% were *M. canettii*.

**Table 5:** Lineage classification of *Mycobacterium tuberculosis complex* isolates from Djibouti. Coll-nomenclature was inferred from whole genome and targeted next-generation sequencing data, Djibouti nationwide drug-resistance survey, 2014–2015.

MTBC Lineage distribution	n	%
Lineage 1 (EAI)	43	20.8
Lineage 2 (Beijing)	4	1.9
Lineage 3 (Delhi/CAS)	47	22.7
Lineage 4 (EAS)	100	48.3
*M. canettii*	13	6.3

Among the rifampicin-resistant cases, the lineage 4 was also the most represented (41.2%), followed by lineage 1 and 3 (26.5% each) and lineage 2 (5.9%). No significant association was observed between specific MTB lineages and development of resistance to rifampicin or MDR-TB (Supplementary Table 1).

**Figure 1:**
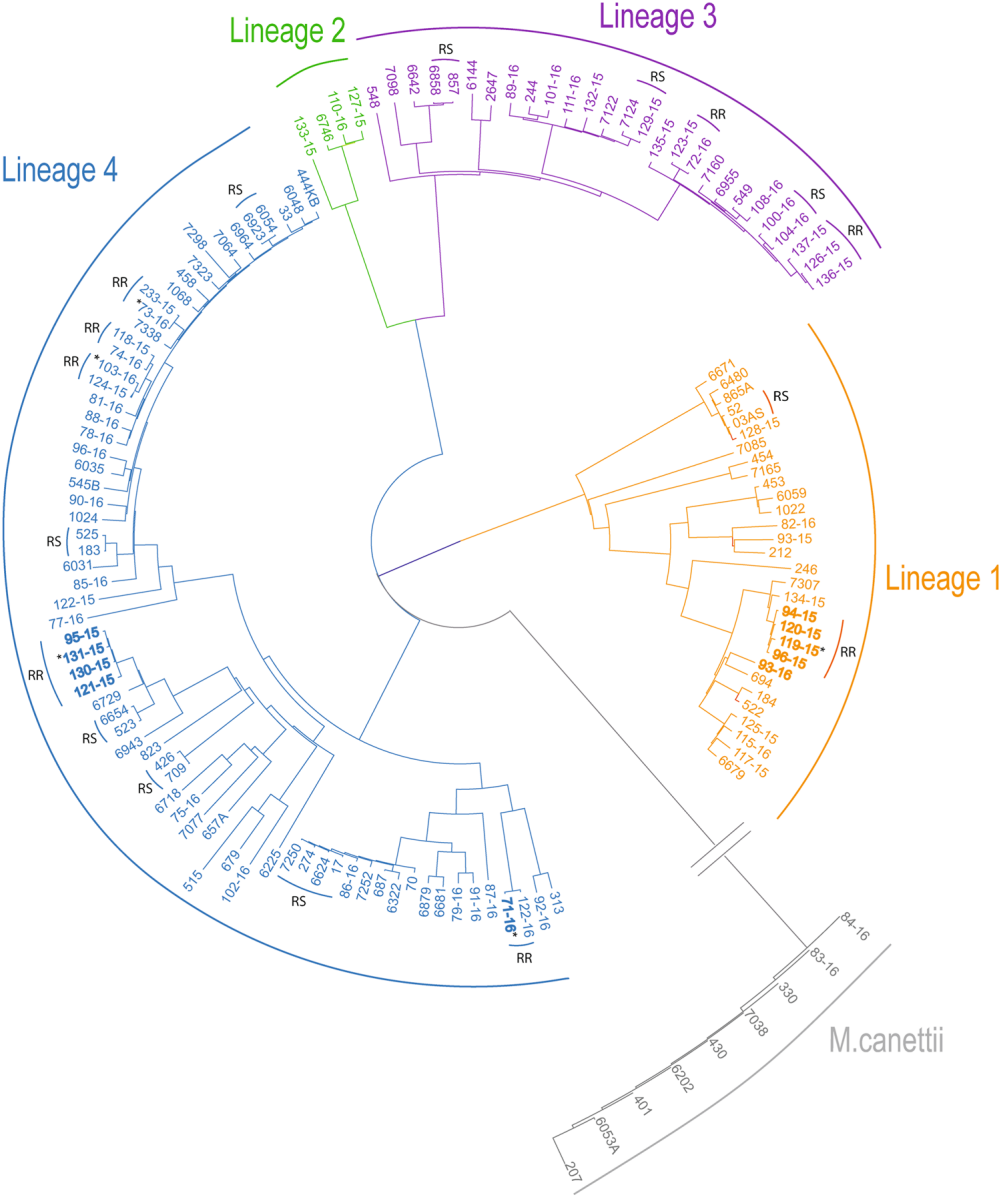
Maximum-likelihood tree of 139 *Mycobacterium tuberculosis complex* isolates from Djibouti. The tree was calculated by using the general time reversible substitution model with gamma distribution and bootstrap resampling based on single-nucleotide polymorphisms identified by whole-genome sequencing. Substitution models were tested and the tree generated by using MetaPiga software version 3.1 and the maximum-likelihood ratio test. Midpoint rooting was performed. Distinct colors were chosen for the lineages identified; the Coll-nomenclature inferred from the whole-genome sequencing data. MDR-TB strains resistant to second line injectable drugs are highlighted in bold. Asterisks indicate clustered MDR-TB isolates from patients aged ≥55 years. Clusters with rifampicin resistant isolates are indicated as RR, while clusters with rifampicin susceptible isolates are indicated as RS. Scale bar indicates nucleotide substitutions per site.

Cluster analysis was performed on a subset of 139 isolates comprising the majority (89.2%) of rifampicin-resistant and 32.1% of rifampicin-susceptible strains (Fig. 1) for which WGS data was available (Supplementary Figure 1). Seventeen whole genome-based clusters, as defined by differences in SNP profiles of less than 12 SNPs, were identified: 8 among rifampicin-resistant and 9 among rifampicin-susceptible strains, including 2 to 4 isolates each. Notably, 21 out of 33 (63.6%) rifampicin-resistant strains analyzed were clustered, into small groups, as opposed to only 20 out of 106 (18.9%) of the rifampicin-susceptible strains. The rifampicin-resistant clusters included 9 out of 14 (64.3%) new TB cases and 12 out of 20 (60%) of previously treated cases. In addition, 4 out of 6 (66.7%) isolates carrying the *rrs* 1401 g mutation which confers resistance to second-line injectable agents were also found in a single cluster, suggesting possible transmission. Similarly, all isolates carrying the specific capreomycin resistance-conferring mutation (Asn236Lys) in the *tlyA* gene belonged to the same cluster (Fig. 1).

## Discussion

In this manuscript, we report the results of the first nationwide TB drug resistance survey conducted in Djibouti following WHO guidelines^14^. Prevalence of MDR-TB (4.7% of new and 30.8% of previously treated TB cases) are significantly higher than expected and higher than those reported in neighboring Ethiopia (2.7% of new and 14% of previously treated) and in other countries of the Eastern Mediterranean region^1^ but comparable to those detected in Somalia (5.2% of new and 40.8% of previously treated TB cases)^8^. These levels of MDR-TB are among the highest ever reported in the African and Eastern Mediterranean regions. Furthermore, results from genotypic drug susceptibility testing show very high frequencies of mutations associated with resistance to pyrazinamide (68%) and second line injectable agents (18% and 29% of isolates resistant to aminoglycosides and to capreomycin, respectively) among the MDR-TB cases.

We found that most cases with rifampicin resistant strains were also MDR, which differs from what has been described in other countries of the region such as Somalia^8^ and Egypt^1^ where rifampicin resistance was significantly higher than MDR-TB. The almost complete overlap between rifampicin resistance and MDR-TB in Djibouti could be explained by the extensive use of fixed dose combination of rifampicin and isoniazid in the treatment of TB in the country^15^. Among MDR-TB cases, the high frequency (>60%) of mutations associated with pyrazinamide resistance in the *pncA* gene is similar to findings observed recently in other settings^16,17^. These data highlight the importance of determining the pyrazinamide susceptibility profile in patients affected by MDR-TB to avoid the use of an ineffective agent in a second-line regimen. In contrast, the very high prevalence of resistance to second-line injectable agents in patients with MDR-TB is an unexpected result, given the controlled use of these medicines by the national TB Control Programme and their unavailability on the market. Conversely, resistance to fluoroquinolones was not detected in patients with rifampicin-resistant TB nor in those with rifampicin-susceptible TB. This finding is rather unexpected as well, given that resistance to fluoroquinolones has been documented repeatedly and regularly in the country (e.g. 7 XDR-TB cases were reported in Djibouti in 2015). Importantly, WGS-based typing analysis showed that all except one of the TB isolates resistant to second-line injectables and to capreomycin belonged to two distinct clusters suggesting that resistance to this class of drugs is due to transmission rather than acquisition during treatment. Similarly, the MDR-TB strains were more frequently clustered (64% belonged to clusters) as opposed to the rifampicin-susceptible ones (18% belonged to clusters). Altogether, these findings represent additional evidence that most of the burden of drug-resistant TB is now the result of transmission rather than acquisition of resistance^18^, with up to 95.9% of MDR-TB in new TB cases and 61.3% in previously treated cases being due to transmission^19^.

Transmission of resistance appears to be involved also in the association of MDR-TB with older age (≥55 years) (OR 3.7). Notably, 6 isolates representing 60% of MDR-TB patients aged ≥55 years were new TB cases and, among those, 83% were found to be clustered into 5 different groups based on whole genome sequencing analysis (Fig. 1). These results thus suggest that transmission, rather than reactivation of previously treated disease, is the driving factor.

In addition, the risk factor analysis showed a strong positive association between MDR-TB and diabetes (OR 9.9). While a growing amount of data from observational studies suggest an association between diabetes mellitus and TB treatment failure, relapse and re-infection^20,21^, its role as risk factor for development or acquisition of MDR-TB remains controversial^22,23^. The altered metabolism of rifampicin in TB patients with diabetes mellitus^24^ may partly explain our finding. Further investigations would be required to better characterize these MDR-TB patients in terms of treatment modality, glycemic control and kidney function. Finally, we found a strong association between MDR-TB and previous history of TB treatment (OR 11.6), which is a common finding in such surveys^8,25^.

In this survey, a broad array of molecular based diagnostic tools was combined to obtain a comprehensive picture of the drug resistant profiles and to describe the phylogeny of the MTB strains circulating in the country. The XpertMTB/RIF assay, used to identify *M. tuberculosis* and to detect rifampicin resistance-associated mutations, is now becoming an integral part of the diagnostic algorithm in TB drug resistance surveys^26,27^ as it drastically reduces the workload at reference laboratories and costs for testing. Moreover, this nationwide survey was the first to use targeted next generation sequencing on DNA extracted directly from sputum specimens. This allowed the detection of mutations associated with resistance to other first line and second line anti-TB drugs in addition to rifampicin without the need of obtaining pure MTB isolates. This is an important advance for this type of surveys that historically have been relying on culture and phenotypic susceptibility testing. The use of sequencing reduces logistic problems related to sample storage and transportation which very often affect the quality of clinical specimens and result into losses of specimens^2^. It is important to note that the drug resistance survey conducted in Somalia^7^ only used first line probe assay thus limiting the detection of resistance to rifampicin and isoniazid. Our results show that the use of a diagnostic tool that allows to provide a more complete drug susceptibility profile would be important to assess whether the worryingly high prevalence of resistance to pyrazinamide and to injectable agents among MDR-TB cases in Djibouti extends to neighboring countries as well.

This study has several limitations. Although the overall number of cases enrolled in the survey reached the target sample size following an extension of the duration of recruitment (7 months), the number of cases recruited in health facilities outside the capital city of Djibouti was only 50% of the number expected (23 cases out of 47). This was due to logistic problems related to the referral of the samples to the reference laboratory and to the fact that two sites outside the city of Djibouti were not functional at the time when the survey took place. This means that cases recruited at the national reference hospital were over-represented in the survey. Secondly, the survey was designed to investigate the proportion of MDR-TB among new and previously treated TB cases and thus it was under-powered to estimate the proportion of resistance to pyrazinamide and second line injectable agents. Lastly, genotypic testing for agents other than rifampicin were only available for slightly less than 60% of the rifampicin-susceptible samples. As a consequence, levels of resistance to isoniazid not associated with rifampicin resistance (i.e. mono- or poly-isoniazid resistance) may not be considered representative of the country situation and were outside the scope of the study design.

Findings from this study have several important implications. They point towards the need of extending rapid susceptibility testing at least to rifampicin to all laboratory confirmed new TB cases, in line with the End TB Strategy calling for universal drug susceptibility testing^28^. The necessity of routinely screening MDR-TB patients for resistance to pyrazinamide, second-line injectable agents and fluoroquinolones is important to inform decisions on best choice of regimen. Coverage for rapid drug susceptibility testing must be expanded to diagnostic centres located outside the city of Djibouti either by strengthening the referral system for the rapid transport of samples towards the TB reference laboratory or by the local use of low-throughput portable molecular based diagnostics. These findings are essential to guide the national TB control program and its partners in the planning and implementation of better targeted public health responses to contain the spread of resistant strains. In particular, urgent action should be taken to strengthen TB and drug resistant TB case detection and to implement measures to control transmission in congregate settings by ensuring prompt and effective treatment.

## Methods

### Study subjects and recruitment

A nationwide drug resistance survey was conducted in Djibouti from September 2014 to March 2015. All new and previously treated pulmonary TB patients of any age which presented to any microscopy center in the country were eligible for enrolment if they were sputum smear-positive on direct microscopy and positive for *M. tuberculosis* complex (MTBC) by Xpert MTB/RIF assay. New sputum smear positive pulmonary TB cases were defined as patients who were never treated before or received anti-TB agents for less than one month, while previously treated smear positive pulmonary TB cases included patients who started a re-treatment regimen after previous treatment had failed, patients who return to treatment after having been lost to follow-up, or patients who had previously been declared as cured or having completed treatment and are diagnosed with bacteriologically positive TB. Consecutive patients meeting the eligibility criteria were enrolled. Informed consent was required ahead of enrolment. A structured questionnaire was completed by all enrolled patients to collect clinical and socio-demographic data.

### Sample size and sampling method

Given the small size of the country and its health care structure, all (17) TB diagnostic facilities in the country were included in the survey. The sample size was calculated based on the total number of new sputum smear positive pulmonary cases registered in the country in 2012 (1194) and the expected prevalence of rifampicin resistance (1.8%) among new TB cases, with an absolute precision of 1.5% at the 95% confidence limit. The sample size was increased by 20% to account for potential losses of samples due to no growth or contamination of culture thus totaling 301 (Supplementary Figure 2). Based on the recent notification rhythm in the country it was estimated that such a survey would take 3 months to complete if all centers were recruiting at 100%; during this time about 66 additional previously treated cases would be expected to present to the treatment centers and would be recruited into the survey.

### Laboratory procedures at TB National Reference Laboratory (NRL)

Two sputum samples were collected from each patient and examined by Ziehl-Neelsen staining at the local diagnostic units. Smear-positive samples were referred to the NRL at the Chakib Saad Hospital in the capital of Djibouti for testing using the Xpert MTB/RIF assay (Cepheid, Sunnyvale, CA, USA). In cases where rifampicin resistance was detected, MTB strains were isolated by liquid culture using the BACTEC^TM^MGIT^TM^ 960 System (MGIT) (BD Bioscience, Erebodegem, Belgium). Positive MTB cultures underwent first-line drug susceptibility testing (DST) in liquid media using the following critical concentrations: rifampicin 1.0 mg/L, isoniazid 0.1 mg/L, ethambutol 5.0 mg/L and streptomycin 1.0 mg/L. All rifampicin-resistant strains and all available rifampicin-susceptible sputum specimens were sent to the TB supranational reference laboratory (SRL) in Milan, Italy for genotypic DST based on next generation sequencing (Supplementary Figure 3). Handling of sputum samples and MTB culture isolates was conducted following essential biosafety measures as recommended by WHO^29^. MTB isolates were shipped to SRL Milan following the IATA regulations for UN2814 Infectious Substances affecting humans (Class 6.2).

### HIV testing

All TB patients enrolled in the survey received rapid HIV testing according to national HIV surveillance policies. All patients positive to rapid HIV testing using Alere Determine™ HIV-1/2 (Alere Medical Co. Ltd) underwent confirmatory testing by ImmunoComb II HIV 1&2 BiSpot (Orgenics Ltd.). Testing was provided in the presence of a counseling service.

### Culture and genotypic drug susceptibility testing at Supranational reference laboratory (SRL)

Sputum samples were sent to SRL Milan using OMNIgene SPUTUM reagent (DNA Genotek) and processed according to manufacturer’s instructions^30^. Decontaminated pellets were inoculated into MGIT tubes for culture. WGS was performed on the available cultured MTB isolates while targeted next generation sequencing was performed on direct specimens when strains could not be retrieved (i.e. culture contaminated or no growth). Supplementary Figure 1 shows the summary of results for the enrolled TB cases. Whole genome or targeted sequencing were performed on all available rifampicin-resistant and rifampicin-susceptible samples.

For WGS, genomic DNA was extracted from cultured isolates using the cetyl trimethylammonium bromide (CTAB) method as previously described^31^. Prior to submission for WGS, DNA was quality assessed and quantified using the Qubit dsDNA BR assay (Life Technologies, Paisley, UK). Paired-end libraries of 100 bp read length were prepared using the Nextera XT DNA Sample Preparation kit (Illumina Inc., San Diego, CA, USA) and sequenced on an Illumina HiSeq 2500 platform according to the manufacturer’s instructions. Downstream analysis was performed using a dedicated in-house bioinformatics pipeline including quality control check, alignment to H37Rv reference genome, recalibration and variant calling. A mean read coverage depth of at least >30x, with at least 4 reads on forward and reverse strand, at least 4 allele calls with base quality ≥20 and allele frequency ≥50%. were considered acceptable to call variants. The association of mutations with drug resistance development was assigned based on the available scientific literature^10,11,32–35^.

For targeted sequencing, DNA was extracted from heat inactivated clinical specimens and amplified using the Deeplex^®^-MycTB assay (Beta version) (GenoScreen) according to manufacturer’s instructions. Amplicons were purified using AMPure^®^ XP (Agencourt^®^) magnetic beads and quantified by Qubit dsDNA BR assay (Life Technologies, Paisley, UK). Libraries for NGS were prepared as described above and sequenced on Illumina MiniSeq platform according to the manufacturer’s instructions. Total variant calling was performed using a dedicated, parameterized software developed by GenoScreen. Supplementary Figure 4 shows the drug resistance associated mutations identified by sequencing.

### MTB lineage and cluster analysis

MTB strain lineage information was assigned according to a list of validated phylogenetic single nucleotide polymorphisms (SNPs) using a dedicated in-house bioinformatics pipeline. Cluster analysis was assessed by SNP identity considering a maximum of 12 variants as threshold to determine whole genome-based clusters indicative of transmission^36^. The phylogenetic tree was calculated using the maximum-likelihood method and the general time reversible (GTR) substitution model, rate heterogeneity, without invariant sites using a gamma distribution as well as bootstrap resampling. Substitution models were tested and trees calculated using the MetaPiga software version 3.1^37^ and the maximum-likelihood ratio test^38^. We applied midpoint rooting with FigTree software version 1.4.3 (http://tree.bio.ed.ac.uk/software/figtree/) and performed formatting by using the online tool Evolview^39^.

### Data management and analysis

A standardized questionnaire was used to collect demographic and clinical information, including TB treatment history, diabetes and HIV status from all the enrolled patients. All data were consolidated by the study coordinator at the National reference laboratory. Statistical analysis was performed by IBM SPSS software (Version 22).

Associations between patient characteristics (socio-demographic or clinical) and drug resistance were explored by binary logistic regression performing both univariate and multivariate analysis. Multivariate analysis was performed using the backward logistic regression method. The magnitude and statistical significance (p < 0.05) of association with potential risk factors were expressed as odd ratios with their 95% confidence intervals (CI).

### Ethics approval

The study protocol of the National drug resistance survey was approved by National Ethical Committee of Djibouti. All methods were performed according to the guidelines described in the study protocol of the National drug resistance survey. Patients signed informed consent. All patients diagnosed with TB and drug-resistant TB received appropriate treatment.

### Data availability

The WGS data generated during the current study are available as fastq files in the Sequence Read Archive of the National Center for Biotechnology Information (accession number SRP111624).

## Electronic supplementary material

Supplementary material
