# Supplementary material for: Culture and Next-generation sequencing-based drug susceptibility testing unveil high levels of drug-resistant-TB in Djibouti: results from the first national survey
Source: Sci Rep. 2017 Dec 15;7:17672. doi: 10.1038/s41598-017-17705-3 (PMC5732159; doi:10.1038/s41598-017-17705-3)
Supplement: Supplementary file 1 — Supplementary material [file 41598_2017_17705_MOESM1_ESM.doc]

**Culture and Next-generation sequencing-based drug susceptibility testing unveil high levels of drug-resistant-TB in Djibouti: results from the first national survey.**

Elisa Tagliani, Mohamed Osman Hassan, Yacine Waberi, Maria Rosaria de Filippo, Dennis Falzon, Anna S. Dean, Matteo Zignol, Philip Supply, Mohamed Ali Abdoulkader, Hawa Hassangue, Daniela Maria Cirillo.

**Supplementary Table 1**. Association between Mycobacterium tuberculosis Complex Phylogenetic Lineage and MDR-TB.

| **Phylogenetic lineage** | **OR (95% CI)** | **p-value** |
| --- | --- | --- |
| Lineage 1 | 1.61 (0.68; 3.8) | 0.27 |
| Lineage 2 | 3.69 (0.59; 23.0) | 0.62 |
| Lineage 3 | 0.97 (0.39; 2.40) | 0.94 |
| Lineage 4 | 0.84 (0.39; 1.81) | 0.66 |

**Supplementary Figure 1**. Summary of results of enrolled TB cases, culture, phenotypic and genotypic drug susceptibility testing.

RIF result available

(n = 367)

Smear positive and Xpert MTB/RIF positive eligible patients (n = 367)

New = 301

Previously treated = 66

Genotypic profiling available (n = 36)

WGS (n = 33)

Targeted sequencing (n = 3)

Genotypic profiling available (n = 191)

WGS (n = 106)

Targeted sequencing (n = 85)

Genotypic profile not available (n = 1)

Genotypic profile not available (n = 139)

RIF resistant (n = 37)

New = 14

Previously treated = 23

RIF susceptible (n = 330)

New = 287

Previously treated = 43

INH resistant (MDR-TB) (n= 34)

New n = 14

Previously treated n = 20

INH resistant (n= 8)

New n = 7

Previously treated n = 1

INH susceptible (n= 2)

New = 0

Previously treated = 2

INH susceptible (n= 183)

New = 166

Previously treated = 17

INH no result (n= 1)

New = 0

Previously treated = 1

INH no result (n= 139)

New = 114

Previously treated = 25

RIF: rifampicin; INH: isoniazid; WGS: whole genome sequencing

**Supplementary Figure 2**. Number or pulmonary TB (PTB) cases notified in Djibouti in 2012 by diagnostic center.

|  |  | **Notification 2012** | | **Sample** | |
| --- | --- | --- | --- | --- | --- |
| **Region** | **Diagnostic center** | **New cases PTB** | **Previously treated PTB** | **New cases PTB** | **Previously treated PTB** |
| Djibouti | Paul Faure | 600 | 186 | 151 | 47 |
| Djibouti | Balbala 1 | 46 | 2 | 12 | 1 |
| Djibouti | Balbala 2 | 111 | 17 | 28 | 4 |
| Djibouti | Arrhiba | 96 | 10 | 24 | 2 |
| Djibouti | PK12 | 20 | 3 | 5 | 1 |
| Djibouti | Eingueila | 20 | 0 | 5 | 0 |
| Djibouti | Farahhad | 43 | 10 | 11 | 3 |
| Djibouti | Ibrahim Balala | 24 | 4 | 6 | 1 |
| Djibouti | Hayableh | 21 | 0 | 5 | 0 |
| Djibouti | Ambouli | 27 | 0 | 7 | 0 |
| Djibouti | Khor Burhan | 20 | 0 | 5 | 0 |
| Djibouti | HGP | 20 | 0 | 5 | 0 |
| Ali Sabieh | Ali Sabieh | 53 | 8 | 13 | 2 |
| Dikhil | Dikhil | 43 | 6 | 11 | 1 |
| Tadjourah | Tadjourah | 13 | 1 | 3 | 0 |
| Tadjourah | Dorra | 22 | 7 | 6 | 2 |
| Obock | Obock | 15 | 8 | 4 | 2 |
|  |  | 1194 | 262 | 301 | 66 |

The sample size was calculated according to the following formula:


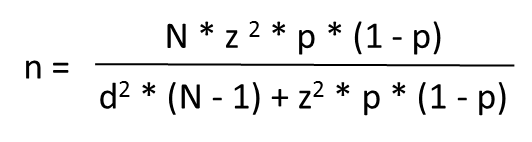


N corresponds to the number of notified cases (1194); z to 95%CI intervals (1,96); d to precision (0,015); p to estimated prevalence of MDR-TB (0,018).

**Supplementary Figure 3**. Laboratory diagnostic algorithm for TB cases enrolled in the survey.

**NRL**

**SRL**

Smear negative

Excluded from the survey

Ziehl-Neelsen microscopy

Send 2 sputum specimens to National reference laboratory

XpertMTB/RIF test on one sputum specimen

Liquid culture and phenotypic drug susceptibility assay to first line anti-TB drugs

Next generation sequencing (whole genome sequencing on MTBC isolates and targeted sequencing on sputum specimens)

MTB not detected

Smear positive

Store specimens at -20°C

MTB detected and rifampicin indeterminate

MTB detected and rifampicin resistant

Storage of second sputum specimens

Culture negative or contaminated

Send rifampicin-resistant isolates and rifampicin-susceptible sputum samples to Supranational reference laboratory

Repeat culture on second specimen

MTB detected and rifampicin susceptible

Store MTBC isolates at -20°C

TB suspect cases

**DISTRICT**

**Supplementary Figure 4**. Database of 367 samples collected over the course of the National Drug Resistance Survey 2014-2015.

|  |  | **Genotypic drug resistance profile^1^** | | | | | | | | | |  |
| --- | --- | --- | --- | --- | --- | --- | --- | --- | --- | --- | --- | --- |
| # |  | **RIF** | **INH** | | **PZA** | | **FQs** | | **SL-INJ** | **KAN** | **CAPREO** | **Accession Number** |
| **Sample ID** | **Xpert**  **MTB/RIF** | ***rpoB*** | ***katG*** | ***inhA*** | ***pncA*** | ***panD*** | ***gyrA*** | ***gyrB*** | ***rrs*** | ***eis*** | ***tlyA*** |
| 96/15 | R | Ser450Trp | Ser315Thr | no mut | Asp158_Stop187del | no mut | no mut | no mut | a1401g | no mut | no mut | SRX2996748 |
| 95/15 | R | Ser450Leu | Ser315Thr | no mut | no mut | no mut | no mut | no mut | no mut | no mut | Asn236Lys | SRX2996747 |
| 94/15 | R | Ser450Trp | Ser315Thr | no mut | Asp158_Stop187del | no mut | no mut | no mut | a1401g | no mut | no mut | SRX2996746 |
| 93/15 | R | Ser450Leu | no mut | c-15t | Met1Thr | no mut | no mut | no mut | a1401g | no mut | no mut | SRX2996744 |
| 74/16 | R | Ser450Leu | Ser315Thr | no mut | His51Pro | no mut | no mut | no mut | no mut | no mut | no mut | SRX2996848 |
| 73/16 | R | Ser450Leu | Ser315Thr | no mut | no mut | no mut | no mut | no mut | no mut | no mut | no mut | SRX2996851 |
| 72/16 | R | Ser450Leu | Ser315Thr | no mut | no mut | no mut | no mut | no mut | no mut | no mut | no mut | SRX2996856 |
| 71/16 | R | Ser450Leu | Ser315Thr | no mut | Asp49Ala | no mut | no mut | no mut | a1401g | no mut | no mut | SRX2996860 |
| 233/15 | R | Ser450Leu | Ser315Thr | no mut | no mut | no mut | no mut | no mut | no mut | no mut | no mut | SRX2996788 |
| 137/15 | R | Ser450Leu | Ser315Thr | no mut | no mut | no mut | no mut | no mut | no mut | no mut | no mut | SRX2996823 |
| 136/15 | R | Ser450Leu | Ser315Thr | no mut | Val125Phe | no mut | no mut | no mut | no mut | no mut | no mut | SRX2996826 |
| 135/15 | R | His445Tyr | no mut | no mut | no mut | no mut | no mut | no mut | no mut | no mut | no mut | SRX2996825 |
| 134/15 | R | Ser450Leu | no mut | c-15t | t-7c | no mut | no mut | no mut | no mut | no mut | no mut | SRX2996828 |
| 133/15 | R | Gln432 fs | Ser315Thr | no mut | no mut | no mut | no mut | no mut | no mut | no mut | no mut | SRX2996827 |
| 132/15 | R | Ser450Leu | Ser315Thr | no mut | no mut | no mut | no mut | no mut | no mut | no mut | no mut | SRX2996809 |
| 131/15 | R | Ser450Leu | Ser315Thr | no mut | Lys96Asn | no mut | no mut | no mut | no mut | no mut | Asn236Lys | SRX2996810 |
| 130/15 | R | Ser450Leu | Ser315Thr | no mut | Lys96Asn | no mut | no mut | no mut | no mut | no mut | Asn236Lys | SRX2996803 |
| 129/15 | R | Ser450Leu | no mut | no mut | no mut | no mut | no mut | no mut | no mut | no mut | no mut | SRX2996804 |
| 128/15 | R | Ser450Leu | no mut | c-15t | no mut | no mut | no mut | no mut | no mut | no mut | no mut | SRX2996801 |
| 127/15 | R | Ser450Leu | Ser315Thr | no mut | Asp8Gly | no mut | no mut | no mut | no mut | no mut | no mut | SRX2996802 |
| 126/15 | R | Ser450Leu | Ser315Thr | no mut | Val125Phe | no mut | no mut | no mut | no mut | no mut | no mut | SRX2996807 |
| 125/15 | R | Ser450Leu | Ser315Thr | no mut | Val131 fs | no mut | no mut | no mut | no mut | no mut | no mut | SRX2996808 |
| 124/15 | R | Ser450Leu | Ser315Thr | no mut | Thr160Ala | no mut | no mut | no mut | no mut | no mut | no mut | SRX2996805 |
| 123/15 | R | Ser450Leu | Ser315Thr | no mut | no mut | no mut | no mut | no mut | no mut | no mut | no mut | SRX2996806 |
| 122/16 | R | Ser450Leu | Ser315Thr | no mut | Asp49Ala | no mut | no mut | no mut | no mut | no mut | no mut | SRX2996864 |
| 122/15 | R | Ser450Leu | Ser315Thr | no mut | Phe58Leu | no mut | no mut | no mut | no mut | no mut | no mut | SRX2996863 |
| 121/15 | R | Ser450Leu | Ser315Thr | no mut | Val128Gly | no mut | no mut | no mut | no mut | no mut | Asn236Lys | SRX2996869 |
| 120/15 | R | Ser450Trp | Ser315Thr | no mut | Asp158_Stop187del | no mut | no mut | no mut | a1401g | no mut | no mut | SRX2996868 |
| 119/15 | R | Ser450Trp | Ser315Thr | no mut | Asp158_Stop187del | no mut | no mut | no mut | a1401g | no mut | no mut | SRX2996867 |
| 118/15 | R | Ser450Leu | Ser315Thr | no mut | Thr76Pro | no mut | no mut | no mut | no mut | no mut | no mut | SRX2996866 |
| 117/15 | R | Asp435Ala + Leu443_Thr444del + His445Pro | Ser315Thr | t-8a | no mut | no mut | no mut | no mut | no mut | no mut | no mut | SRX2996873 |
| 111/16 | R | Asp435Val | Ser539 fs | no mut | Val139Met | no mut | no mut | no mut | no mut | no mut | no mut | SRX2996841 |
| 103/16 | R | Ser450Leu | Ser315Thr | no mut | Thr160Ala | no mut | no mut | no mut | no mut | no mut | no mut | SRX2996862 |
| 96/16 | S | no mut | no mut | no mut | no mut | no mut | no mut | no mut | no mut | no mut | no mut | SRX2996740 |
| 93/16 | S | no mut | no mut | no mut | no mut | no mut | no mut | no mut | no mut | no mut | no mut | SRX2996745 |
| 92/16 | S | no mut | no mut | no mut | no mut | no mut | no mut | no mut | no mut | no mut | no mut | SRX2996743 |
| 91/16 | S | no mut | no mut | no mut | no mut | no mut | no mut | no mut | no mut | no mut | no mut | SRX2996742 |
| 90/16 | S | no mut | no mut | no mut | no mut | no mut | no mut | no mut | no mut | no mut | no mut | SRX2996741 |
| 89/16 | S | no mut | no mut | no mut | no mut | no mut | no mut | no mut | no mut | no mut | no mut | SRX2996769 |
| 88/16 | S | no mut | no mut | no mut | no mut | no mut | no mut | no mut | no mut | no mut | no mut | SRX2996770 |
| 87/16 | S | no mut | no mut | no mut | no mut | no mut | no mut | no mut | no mut | no mut | no mut | SRX2996761 |
| 865A | S | no mut | no mut | no mut | no mut | no mut | no mut | no mut | no mut | no mut | no mut | SRX2996763 |
| 86/16 | S | no mut | no mut | no mut | no mut | no mut | no mut | no mut | no mut | no mut | no mut | SRX2996762 |
| 85/16 | S | no mut | no mut | no mut | no mut | no mut | no mut | no mut | no mut | no mut | no mut | SRX2996874 |
| 84/16 | S | no mut | no mut | no mut | no mut | Met117Thr | no mut | no mut | no mut | no mut | no mut | SRX2996876 |
| 83/16 | S | no mut | no mut | no mut | no mut | Met117Thr | no mut | no mut | no mut | no mut | no mut | SRX2996767 |
| 82/16 | S | no mut | no mut | no mut | no mut | no mut | no mut | no mut | no mut | no mut | no mut | SRX2996768 |
| 81/16 | S | no mut | no mut | c-15t | no mut | no mut | no mut | no mut | no mut | no mut | no mut | SRX2996842 |
| 79/16 | S | no mut | no mut | no mut | no mut | no mut | no mut | no mut | no mut | no mut | no mut | SRX2996845 |
| 78/16 | S | no mut | no mut | no mut | no mut | no mut | no mut | no mut | no mut | no mut | no mut | SRX2996844 |
| 77/16 | S | no mut | no mut | no mut | no mut | no mut | no mut | no mut | no mut | no mut | no mut | SRX2996847 |
| 75/16 | S | no mut | no mut | no mut | no mut | no mut | no mut | no mut | no mut | no mut | no mut | SRX2996849 |
| 7077 | S | no mut | no mut | no mut | no mut | no mut | no mut | no mut | no mut | no mut | no mut | SRX2996846 |
| 657A | S | no mut | no mut | no mut | no mut | no mut | no mut | no mut | no mut | no mut | no mut | SRX2996779 |
| 6053A | S | no mut | no mut | no mut | no mut | Met117Thr | no mut | no mut | no mut | no mut | no mut | [SRX2996758](https://www.ncbi.nlm.nih.gov/sra/SRX2996758%5Baccn%5D) |
| 548 | S | no mut | no mut | no mut | no mut | no mut | no mut | no mut | no mut | no mut | no mut | SRX2996751 |
| 545B | S | no mut | no mut | no mut | no mut | no mut | no mut | no mut | no mut | no mut | no mut | SRX2996752 |
| 453KB | S | no mut | no mut | no mut | no mut | no mut | no mut | no mut | no mut | no mut | no mut | SRX2996793 |
| 444KB | S | no mut | no mut | no mut | no mut | no mut | no mut | no mut | no mut | no mut | no mut | SRX2996792 |
| 401 | S | no mut | no mut | no mut | no mut | Met117Thr | no mut | no mut | no mut | no mut | no mut | SRX2996790 |
| 330 | S | no mut | no mut | no mut | no mut | Met117Thr | no mut | no mut | no mut | no mut | no mut | SRX2996782 |
| 33 | S | no mut | no mut | no mut | no mut | no mut | no mut | no mut | no mut | no mut | no mut | SRX2996781 |
| 313 | S | no mut | no mut | no mut | no mut | no mut | no mut | no mut | no mut | no mut | no mut | SRX2996783 |
| 2647 | S | no mut | no mut | no mut | no mut | no mut | no mut | no mut | no mut | no mut | no mut | SRX2996785 |
| 115/16 | S | no mut | no mut | no mut | no mut | no mut | no mut | no mut | no mut | no mut | no mut | SRX2996872 |
| 110/16 | S | no mut | no mut | no mut | no mut | no mut | no mut | no mut | no mut | no mut | no mut | SRX2996870 |
| 108/16 | S | no mut | no mut | no mut | no mut | no mut | no mut | no mut | no mut | no mut | no mut | SRX2996760 |
| 104/16 | S | no mut | no mut | no mut | no mut | no mut | no mut | no mut | no mut | no mut | no mut | SRX2996865 |
| 102/16 | S | no mut | Ser315Thr | no mut | no mut | no mut | no mut | no mut | no mut | no mut | no mut | SRX2996764 |
| 101/16 | S | no mut | no mut | c-15t | no mut | no mut | no mut | no mut | no mut | no mut | no mut | SRX2996765 |
| 100/16 | S | no mut | no mut | no mut | no mut | no mut | no mut | no mut | no mut | no mut | no mut | SRX2996739 |
| 03AS | S | no mut | no mut | no mut | no mut | no mut | no mut | no mut | no mut | no mut | no mut | SRX2996738 |
| 7338 | S | no mut | no mut | no mut | no mut | no mut | no mut | no mut | no mut | no mut | no mut | SRX2996850 |
| 7323 | S | no mut | no mut | no mut | no mut | no mut | no mut | no mut | no mut | no mut | no mut | SRX2996852 |
| 7307 | S | no mut | no mut | no mut | no mut | no mut | no mut | no mut | no mut | no mut | no mut | SRX2996853 |
| 7298 | S | no mut | no mut | no mut | no mut | no mut | no mut | no mut | no mut | no mut | no mut | SRX2996857 |
| 7252 | S | no mut | no mut | no mut | no mut | no mut | no mut | no mut | no mut | no mut | no mut | SRX2996854 |
| 7250 | S | no mut | no mut | no mut | no mut | no mut | no mut | no mut | no mut | no mut | no mut | SRX2996855 |
| 7165 | S | no mut | no mut | no mut | no mut | no mut | no mut | no mut | no mut | no mut | no mut | SRX2996861 |
| 7160 | S | no mut | no mut | no mut | no mut | no mut | no mut | no mut | no mut | no mut | no mut | SRX2996858 |
| 7124 | S | no mut | no mut | no mut | no mut | no mut | no mut | no mut | no mut | no mut | no mut | SRX2996859 |
| 7122 | S | no mut | no mut | no mut | no mut | no mut | no mut | no mut | no mut | no mut | no mut | SRX2996840 |
| 7098 | S | no mut | no mut | no mut | no mut | no mut | no mut | no mut | no mut | no mut | no mut | SRX2996833 |
| 7085 | S | no mut | no mut | no mut | His43Tyr | no mut | no mut | no mut | no mut | no mut | no mut | SRX2996832 |
| 7064 | S | no mut | no mut | no mut | no mut | no mut | no mut | no mut | no mut | no mut | no mut | SRX2996831 |
| 7038 | S | no mut | no mut | no mut | no mut | Met117Thr | no mut | no mut | no mut | no mut | no mut | SRX2996838 |
| 6964 | S | no mut | no mut | no mut | no mut | no mut | no mut | no mut | no mut | no mut | no mut | SRX2996837 |
| 6955 | S | no mut | no mut | no mut | no mut | no mut | no mut | no mut | no mut | no mut | no mut | SRX2996836 |
| 6943 | S | no mut | no mut | no mut | no mut | no mut | no mut | no mut | no mut | no mut | Asn236Lys | SRX2996811 |
| 6923 | S | no mut | no mut | no mut | no mut | no mut | no mut | no mut | no mut | no mut | no mut | SRX2996812 |
| 6879 | S | no mut | no mut | no mut | no mut | no mut | no mut | no mut | no mut | no mut | no mut | SRX2996818 |
| 6858 | S | no mut | no mut | no mut | no mut | no mut | no mut | no mut | no mut | no mut | no mut | SRX2996819 |
| 6746 | S | no mut | no mut | no mut | no mut | no mut | no mut | no mut | no mut | no mut | no mut | SRX2996813 |
| 6729 | S | no mut | no mut | no mut | no mut | no mut | no mut | no mut | no mut | no mut | Asn236Lys | SRX2996814 |
| 6718 | S | no mut | no mut | no mut | no mut | no mut | no mut | no mut | no mut | no mut | no mut | SRX2996815 |
| 6681 | S | no mut | no mut | no mut | no mut | no mut | no mut | no mut | no mut | no mut | no mut | SRX2996816 |
| 6679 | S | no mut | no mut | no mut | no mut | no mut | no mut | no mut | no mut | no mut | no mut | SRX2996772 |
| 6671 | S | no mut | no mut | no mut | no mut | no mut | no mut | no mut | no mut | no mut | no mut | SRX2996771 |
| 6654 | S | no mut | no mut | no mut | no mut | no mut | no mut | no mut | no mut | no mut | Asn236Lys | SRX2996778 |
| 6642 | S | no mut | no mut | no mut | no mut | no mut | no mut | no mut | no mut | no mut | no mut | SRX2996777 |
| 6624 | S | no mut | no mut | no mut | no mut | no mut | no mut | no mut | no mut | no mut | no mut | SRX2996780 |
| 6480 | S | no mut | no mut | no mut | no mut | no mut | no mut | no mut | no mut | no mut | no mut | SRX2996774 |
| 6322 | S | no mut | no mut | no mut | no mut | no mut | no mut | no mut | no mut | no mut | no mut | SRX2996773 |
| 6225 | S | no mut | no mut | no mut | no mut | no mut | no mut | no mut | no mut | no mut | no mut | SRX2996776 |
| 6202 | S | no mut | no mut | no mut | no mut | Met117Thr | no mut | no mut | no mut | no mut | no mut | SRX2996775 |
| 6144 | S | no mut | no mut | no mut | no mut | no mut | no mut | no mut | no mut | no mut | no mut | SRX2996749 |
| 6059 | S | no mut | no mut | no mut | no mut | no mut | no mut | no mut | no mut | no mut | no mut | SRX2996750 |
| 6054 | S | no mut | no mut | no mut | no mut | no mut | no mut | no mut | no mut | no mut | no mut | SRX2996757 |
| 6048 | S | no mut | no mut | no mut | no mut | no mut | no mut | no mut | no mut | no mut | no mut | SRX2996755 |
| 6035 | S | no mut | no mut | no mut | no mut | no mut | no mut | no mut | no mut | no mut | no mut | SRX2996756 |
| 6031 | S | no mut | no mut | no mut | no mut | no mut | no mut | no mut | no mut | no mut | no mut | SRX2996753 |
| 1105 | S | no mut | no mut | no mut | no mut | no mut | no mut | no mut | no mut | no mut | no mut | SRX2996822 |
| 1068 | S | no mut | no mut | no mut | no mut | no mut | no mut | no mut | no mut | no mut | no mut | SRX2996759 |
| 1024 | S | no mut | no mut | no mut | no mut | no mut | no mut | no mut | no mut | no mut | no mut | SRX2996871 |
| 1022 | S | no mut | no mut | no mut | no mut | no mut | no mut | no mut | no mut | no mut | no mut | SRX2996766 |
| 857 | S | no mut | no mut | no mut | no mut | no mut | no mut | no mut | no mut | no mut | no mut | SRX2996875 |
| 823 | S | no mut | no mut | no mut | no mut | no mut | no mut | no mut | no mut | no mut | no mut | SRX2996843 |
| 709 | S | no mut | no mut | no mut | no mut | no mut | no mut | no mut | no mut | no mut | no mut | SRX2996834 |
| 694 | S | no mut | no mut | no mut | no mut | no mut | no mut | no mut | no mut | no mut | no mut | SRX2996835 |
| 687 | S | no mut | no mut | no mut | no mut | no mut | no mut | no mut | no mut | no mut | no mut | SRX2996817 |
| 679 | S | no mut | no mut | no mut | no mut | no mut | no mut | no mut | no mut | no mut | no mut | SRX2996820 |
| 549 | S | no mut | no mut | no mut | no mut | no mut | no mut | no mut | no mut | no mut | no mut | SRX2996754 |
| 525 | S | no mut | no mut | no mut | no mut | no mut | no mut | no mut | no mut | no mut | no mut | SRX2996799 |
| 523 | S | no mut | no mut | no mut | no mut | no mut | no mut | no mut | no mut | no mut | Asn236Lys | SRX2996798 |
| 522 | S | no mut | no mut | no mut | no mut | no mut | no mut | no mut | no mut | no mut | no mut | SRX2996797 |
| 515 | S | no mut | no mut | no mut | no mut | no mut | no mut | no mut | no mut | no mut | no mut | SRX2996796 |
| 458 | S | no mut | no mut | no mut | no mut | no mut | no mut | no mut | no mut | no mut | no mut | SRX2996795 |
| 454 | S | no mut | no mut | no mut | no mut | no mut | no mut | no mut | no mut | no mut | no mut | [SRX2996794](https://www.ncbi.nlm.nih.gov/sra/SRX2996794%5Baccn%5D) |
| 430 | S | no mut | no mut | no mut | no mut | Met117Thr | no mut | no mut | no mut | no mut | no mut | SRX2996791 |
| 426 | S | no mut | no mut | no mut | no mut | no mut | no mut | no mut | no mut | no mut | no mut | SRX2996789 |
| 274 | S | no mut | no mut | no mut | no mut | no mut | no mut | no mut | no mut | no mut | no mut | SRX2996784 |
| 246 | S | no mut | no mut | no mut | no mut | no mut | no mut | no mut | no mut | no mut | no mut | SRX2996786 |
| 244 | S | no mut | no mut | no mut | no mut | no mut | no mut | no mut | no mut | no mut | no mut | SRX2996787 |
| 212 | S | no mut | no mut | c-15t | no mut | no mut | no mut | no mut | no mut | no mut | no mut | SRX2996830 |
| 207 | S | no mut | no mut | no mut | no mut | Met117Thr | no mut | no mut | no mut | no mut | no mut | SRX2996829 |
| 183 | S | no mut | no mut | no mut | no mut | no mut | no mut | no mut | no mut | no mut | no mut | SRX2996821 |
| 70 | S | no mut | no mut | no mut | no mut | no mut | no mut | no mut | no mut | no mut | no mut | SRX2996839 |
| 52 | S | no mut | no mut | no mut | no mut | no mut | no mut | no mut | no mut | no mut | no mut | SRX2996800 |
| 17 | S | no mut | no mut | no mut | no mut | no mut | no mut | no mut | no mut | no mut | no mut | SRX2996824 |
| 578N | R | Ser450Leu | Ser315Thr | c-15t | Val128Gly | n.a. | no mut | no mut | no mut | no mut | no mut | n.a. |
| 126 | R | Ser450Leu | Ser315Thr | c-15t | Asp8Gly | n.a. | no mut | no mut | no mut | no mut | no mut | n.a. |
| 433 | S | Ser450Leu | Ser315Thr | no mut | no mut | n.a. | no mut | no mut | no mut | no mut | no mut | n.a. |
| 7302N | S | no mut | no mut | no mut | no mut | n.a. | no mut | no mut | no mut | no mut | no mut | n.a. |
| 7112N | S | no mut | no mut | no mut | no mut | n.a. | no mut | no mut | no mut | no mut | no mut | n.a. |
| 6957N | S | no mut | no mut | no mut | no mut | n.a. | no mut | no mut | no mut | no mut | no mut | n.a. |
| 6899N | S | no mut | no mut | no mut | no mut | n.a. | no mut | no mut | no mut | no mut | no mut | n.a. |
| 6749N | S | no mut | no mut | no mut | no mut | n.a. | no mut | no mut | no mut | no mut | no mut | n.a. |
| 6594N | S | no mut | no mut | no mut | no mut | n.a. | no mut | no mut | no mut | no mut | no mut | n.a. |
| 6553N | S | no mut | no mut | no mut | no mut | n.a. | no mut | no mut | no mut | no mut | no mut | n.a. |
| 6503N | S | no mut | Ser315Thr | no mut | no mut | n.a. | no mut | no mut | no mut | no mut | no mut | n.a. |
| 6165N | S | no mut | no mut | no mut | no mut | n.a. | no mut | no mut | no mut | no mut | no mut | n.a. |
| 5706N | S | no mut | no mut | no mut | no mut | n.a. | no mut | no mut | no mut | no mut | no mut | n.a. |
| 49GX | S | no mut | no mut | no mut | no mut | n.a. | no mut | no mut | no mut | no mut | no mut | n.a. |
| 491N | S | no mut | no mut | no mut | no mut | n.a. | no mut | no mut | no mut | no mut | no mut | n.a. |
| 454KB | S | no mut | Ser315Thr | no mut | no mut | n.a. | no mut | no mut | no mut | no mut | no mut | n.a. |
| 436N | S | no mut | no mut | no mut | no mut | n.a. | no mut | no mut | no mut | no mut | no mut | n.a. |
| 397N | S | no mut | no mut | no mut | no mut | n.a. | no mut | no mut | no mut | no mut | no mut | n.a. |
| 387N | S | no mut | Ser315Thr | no mut | no mut | n.a. | no mut | no mut | no mut | no mut | no mut | n.a. |
| 365N | S | no mut | no mut | no mut | no mut | n.a. | no mut | no mut | no mut | no mut | no mut | n.a. |
| 293N | S | no mut | no mut | no mut | no mut | n.a. | no mut | no mut | no mut | no mut | no mut | n.a. |
| 7321 | S | no mut | no mut | no mut | no mut | n.a. | no mut | no mut | no mut | no mut | no mut | n.a. |
| 7287 | S | no mut | no mut | no mut | no mut | n.a. | no mut | no mut | no mut | no mut | no mut | n.a. |
| 7281 | S | no mut | no mut | no mut | no mut | n.a. | no mut | no mut | no mut | no mut | no mut | n.a. |
| 7253 | S | no mut | no mut | no mut | no mut | n.a. | no mut | no mut | no mut | no mut | no mut | n.a. |
| 7192 | S | no mut | no mut | no mut | no mut | n.a. | no mut | no mut | no mut | no mut | no mut | n.a. |
| 7155 | S | no mut | no mut | no mut | no mut | n.a. | no mut | no mut | no mut | no mut | no mut | n.a. |
| 7117 | S | no mut | no mut | no mut | no mut | n.a. | no mut | no mut | no mut | no mut | no mut | n.a. |
| 7026 | S | no mut | no mut | no mut | no mut | n.a. | no mut | no mut | no mut | no mut | no mut | n.a. |
| 7009 | S | no mut | no mut | no mut | no mut | n.a. | no mut | no mut | no mut | no mut | no mut | n.a. |
| 7001 | S | no mut | no mut | no mut | no mut | n.a. | no mut | no mut | no mut | no mut | no mut | n.a. |
| 6998 | S | no mut | no mut | no mut | no mut | n.a. | no mut | no mut | no mut | no mut | no mut | n.a. |
| 6930 | S | no mut | no mut | no mut | no mut | n.a. | no mut | no mut | no mut | no mut | no mut | n.a. |
| 6893 | S | no mut | no mut | no mut | no mut | n.a. | no mut | no mut | no mut | no mut | no mut | n.a. |
| 6827 | S | no mut | no mut | no mut | no mut | n.a. | no mut | no mut | no mut | no mut | no mut | n.a. |
| 6819 | S | no mut | no mut | no mut | no mut | n.a. | no mut | no mut | no mut | no mut | no mut | n.a. |
| 6778 | S | no mut | no mut | no mut | no mut | n.a. | no mut | no mut | no mut | no mut | no mut | n.a. |
| 6740 | S | no mut | no mut | no mut | no mut | n.a. | no mut | no mut | no mut | no mut | Asn236Lys | n.a. |
| 6691 | S | no mut | no mut | no mut | no mut | n.a. | no mut | no mut | no mut | no mut | no mut | n.a. |
| 6496 | S | no mut | no mut | no mut | no mut | n.a. | no mut | no mut | no mut | no mut | no mut | n.a. |
| 6464 | S | no mut | no mut | no mut | no mut | n.a. | no mut | no mut | no mut | no mut | no mut | n.a. |
| 6436 | S | no mut | no mut | no mut | no mut | n.a. | no mut | no mut | no mut | no mut | no mut | n.a. |
| 6368 | S | no mut | no mut | no mut | no mut | n.a. | no mut | no mut | no mut | no mut | no mut | n.a. |
| 6332 | S | no mut | no mut | no mut | no mut | n.a. | no mut | no mut | no mut | no mut | no mut | n.a. |
| 6157 | S | no mut | no mut | no mut | no mut | n.a. | no mut | no mut | no mut | no mut | Asn236Lys | n.a. |
| 6145 | S | no mut | no mut | no mut | no mut | n.a. | no mut | no mut | no mut | no mut | no mut | n.a. |
| 6117 | S | no mut | no mut | no mut | no mut | n.a. | no mut | no mut | no mut | no mut | no mut | n.a. |
| 6096 | S | no mut | no mut | no mut | no mut | n.a. | no mut | no mut | no mut | no mut | no mut | n.a. |
| 6092 | S | no mut | no mut | no mut | no mut | n.a. | no mut | no mut | no mut | no mut | no mut | n.a. |
| 6046 | S | no mut | no mut | no mut | no mut | n.a. | no mut | no mut | no mut | no mut | no mut | n.a. |
| 6042 | S | no mut | no mut | no mut | no mut | n.a. | no mut | no mut | no mut | no mut | no mut | n.a. |
| 5875 | S | no mut | Ser315Thr | no mut | no mut | n.a. | no mut | no mut | no mut | no mut | no mut | n.a. |
| 1299 | S | no mut | no mut | no mut | no mut | n.a. | no mut | no mut | no mut | no mut | no mut | n.a. |
| 1291 | S | no mut | no mut | no mut | no mut | n.a. | no mut | no mut | no mut | no mut | no mut | n.a. |
| 1279 | S | no mut | no mut | no mut | no mut | n.a. | no mut | no mut | no mut | no mut | no mut | n.a. |
| 1274 | S | no mut | no mut | no mut | no mut | n.a. | no mut | no mut | no mut | no mut | no mut | n.a. |
| 1265 | S | no mut | no mut | no mut | no mut | n.a. | no mut | no mut | no mut | no mut | no mut | n.a. |
| 1232 | S | no mut | no mut | no mut | no mut | n.a. | no mut | no mut | no mut | no mut | no mut | n.a. |
| 1086 | S | no mut | no mut | no mut | no mut | n.a. | no mut | no mut | no mut | no mut | no mut | n.a. |
| 1080 | S | no mut | no mut | no mut | no mut | n.a. | no mut | no mut | no mut | no mut | no mut | n.a. |
| 776 | S | no mut | no mut | no mut | no mut | n.a. | no mut | no mut | no mut | no mut | no mut | n.a. |
| 710 | S | no mut | no mut | no mut | no mut | n.a. | no mut | no mut | no mut | no mut | no mut | n.a. |
| 654 | S | no mut | no mut | no mut | no mut | n.a. | no mut | no mut | no mut | no mut | no mut | n.a. |
| 576 | S | no mut | no mut | no mut | no mut | n.a. | no mut | no mut | no mut | no mut | no mut | n.a. |
| 573 | S | no mut | no mut | no mut | no mut | n.a. | no mut | no mut | no mut | no mut | no mut | n.a. |
| 530 | S | no mut | no mut | no mut | no mut | n.a. | no mut | no mut | no mut | no mut | no mut | n.a. |
| 496 | S | no mut | no mut | no mut | no mut | n.a. | no mut | no mut | no mut | no mut | no mut | n.a. |
| 466 | S | no mut | Ser315Thr | no mut | no mut | n.a. | no mut | no mut | no mut | no mut | no mut | n.a. |
| 462 | S | no mut | no mut | no mut | no mut | n.a. | no mut | no mut | no mut | no mut | no mut | n.a. |
| 444 | S | no mut | no mut | no mut | no mut | n.a. | no mut | no mut | no mut | no mut | no mut | n.a. |
| 437 | S | no mut | no mut | no mut | no mut | n.a. | no mut | no mut | no mut | no mut | no mut | n.a. |
| 431 | S | no mut | no mut | no mut | no mut | n.a. | no mut | no mut | no mut | no mut | no mut | n.a. |
| 331 | S | no mut | no mut | no mut | no mut | n.a. | no mut | no mut | no mut | no mut | no mut | n.a. |
| 273 | S | no mut | no mut | no mut | no mut | n.a. | no mut | no mut | no mut | no mut | no mut | n.a. |
| 252 | S | no mut | no mut | no mut | no mut | n.a. | no mut | no mut | no mut | no mut | no mut | n.a. |
| 241 | S | no mut | no mut | no mut | no mut | n.a. | no mut | no mut | no mut | no mut | no mut | n.a. |
| 215 | S | no mut | no mut | no mut | no mut | n.a. | no mut | no mut | no mut | no mut | no mut | n.a. |
| 213 | S | no mut | no mut | no mut | no mut | n.a. | no mut | no mut | no mut | no mut | no mut | n.a. |
| 178 | S | no mut | no mut | no mut | no mut | n.a. | no mut | no mut | no mut | no mut | no mut | n.a. |
| 171 | S | no mut | no mut | no mut | no mut | n.a. | no mut | no mut | no mut | no mut | no mut | n.a. |
| 139 | S | no mut | no mut | no mut | no mut | n.a. | no mut | no mut | no mut | no mut | no mut | n.a. |
| 115 | S | no mut | no mut | no mut | no mut | n.a. | no mut | no mut | no mut | no mut | no mut | n.a. |
| 108 | S | no mut | no mut | no mut | no mut | n.a. | no mut | no mut | no mut | no mut | no mut | n.a. |
| 85 | S | no mut | no mut | no mut | no mut | n.a. | no mut | no mut | no mut | no mut | no mut | n.a. |
| 55 | S | no mut | no mut | no mut | no mut | n.a. | no mut | no mut | no mut | no mut | no mut | n.a. |
| 30 | S | no mut | no mut | no mut | no mut | n.a. | no mut | no mut | no mut | no mut | no mut | n.a. |
| 24 | S | no mut | no mut | no mut | no mut | n.a. | no mut | no mut | no mut | no mut | no mut | n.a. |
| 19 | S | no mut | no mut | no mut | no mut | n.a. | no mut | no mut | no mut | no mut | no mut | n.a. |
| 161 | R | n.a. | n.a. | n.a. | n.a. | n.a. | n.a. | n.a. | n.a. | n.a. | n.a. | n.a. |
| 547 | S | n.a. | n.a. | n.a. | n.a. | n.a. | n.a. | n.a. | n.a. | n.a. | n.a. | n.a. |
| 5625 | S | n.a. | n.a. | n.a. | n.a. | n.a. | n.a. | n.a. | n.a. | n.a. | n.a. | n.a. |
| 5456 | S | n.a. | n.a. | n.a. | n.a. | n.a. | n.a. | n.a. | n.a. | n.a. | n.a. | n.a. |
| 1117 | S | n.a. | n.a. | n.a. | n.a. | n.a. | n.a. | n.a. | n.a. | n.a. | n.a. | n.a. |
| 408 | S | n.a. | n.a. | n.a. | n.a. | n.a. | n.a. | n.a. | n.a. | n.a. | n.a. | n.a. |
| 5910 | S | n.a. | n.a. | n.a. | n.a. | n.a. | n.a. | n.a. | n.a. | n.a. | n.a. | n.a. |
| 03DK | S | n.a. | n.a. | n.a. | n.a. | n.a. | n.a. | n.a. | n.a. | n.a. | n.a. | n.a. |
| 349 | S | n.a. | n.a. | n.a. | n.a. | n.a. | n.a. | n.a. | n.a. | n.a. | n.a. | n.a. |
| 5451 | S | n.a. | n.a. | n.a. | n.a. | n.a. | n.a. | n.a. | n.a. | n.a. | n.a. | n.a. |
| 234 | S | n.a. | n.a. | n.a. | n.a. | n.a. | n.a. | n.a. | n.a. | n.a. | n.a. | n.a. |
| 5809 | S | n.a. | n.a. | n.a. | n.a. | n.a. | n.a. | n.a. | n.a. | n.a. | n.a. | n.a. |
| 279 | S | n.a. | n.a. | n.a. | n.a. | n.a. | n.a. | n.a. | n.a. | n.a. | n.a. | n.a. |
| 6003 | S | n.a. | n.a. | n.a. | n.a. | n.a. | n.a. | n.a. | n.a. | n.a. | n.a. | n.a. |
| 1188 | S | n.a. | n.a. | n.a. | n.a. | n.a. | n.a. | n.a. | n.a. | n.a. | n.a. | n.a. |
| 526 | S | n.a. | n.a. | n.a. | n.a. | n.a. | n.a. | n.a. | n.a. | n.a. | n.a. | n.a. |
| 5958 | S | n.a. | n.a. | n.a. | n.a. | n.a. | n.a. | n.a. | n.a. | n.a. | n.a. | n.a. |
| 648 | S | n.a. | n.a. | n.a. | n.a. | n.a. | n.a. | n.a. | n.a. | n.a. | n.a. | n.a. |
| 5878 | S | n.a. | n.a. | n.a. | n.a. | n.a. | n.a. | n.a. | n.a. | n.a. | n.a. | n.a. |
| 1OBK | S | n.a. | n.a. | n.a. | n.a. | n.a. | n.a. | n.a. | n.a. | n.a. | n.a. | n.a. |
| 01DK | S | n.a. | n.a. | n.a. | n.a. | n.a. | n.a. | n.a. | n.a. | n.a. | n.a. | n.a. |
| 5917 | S | n.a. | n.a. | n.a. | n.a. | n.a. | n.a. | n.a. | n.a. | n.a. | n.a. | n.a. |
| 211 | S | n.a. | n.a. | n.a. | n.a. | n.a. | n.a. | n.a. | n.a. | n.a. | n.a. | n.a. |
| 803 | S | n.a. | n.a. | n.a. | n.a. | n.a. | n.a. | n.a. | n.a. | n.a. | n.a. | n.a. |
| 1 | S | n.a. | n.a. | n.a. | n.a. | n.a. | n.a. | n.a. | n.a. | n.a. | n.a. | n.a. |
| 6008 | S | n.a. | n.a. | n.a. | n.a. | n.a. | n.a. | n.a. | n.a. | n.a. | n.a. | n.a. |
| 5510 | S | n.a. | n.a. | n.a. | n.a. | n.a. | n.a. | n.a. | n.a. | n.a. | n.a. | n.a. |
| 6319 | S | n.a. | n.a. | n.a. | n.a. | n.a. | n.a. | n.a. | n.a. | n.a. | n.a. | n.a. |
| 5747 | S | n.a. | n.a. | n.a. | n.a. | n.a. | n.a. | n.a. | n.a. | n.a. | n.a. | n.a. |
| 02DK | S | n.a. | n.a. | n.a. | n.a. | n.a. | n.a. | n.a. | n.a. | n.a. | n.a. | n.a. |
| 5911 | S | n.a. | n.a. | n.a. | n.a. | n.a. | n.a. | n.a. | n.a. | n.a. | n.a. | n.a. |
| 1011 | S | n.a. | n.a. | n.a. | n.a. | n.a. | n.a. | n.a. | n.a. | n.a. | n.a. | n.a. |
| 441 | S | n.a. | n.a. | n.a. | n.a. | n.a. | n.a. | n.a. | n.a. | n.a. | n.a. | n.a. |
| 5453 | S | n.a. | n.a. | n.a. | n.a. | n.a. | n.a. | n.a. | n.a. | n.a. | n.a. | n.a. |
| 1062 | S | n.a. | n.a. | n.a. | n.a. | n.a. | n.a. | n.a. | n.a. | n.a. | n.a. | n.a. |
| 6179 | S | n.a. | n.a. | n.a. | n.a. | n.a. | n.a. | n.a. | n.a. | n.a. | n.a. | n.a. |
| 988 | S | n.a. | n.a. | n.a. | n.a. | n.a. | n.a. | n.a. | n.a. | n.a. | n.a. | n.a. |
| 1176 | S | n.a. | n.a. | n.a. | n.a. | n.a. | n.a. | n.a. | n.a. | n.a. | n.a. | n.a. |
| 5928 | S | n.a. | n.a. | n.a. | n.a. | n.a. | n.a. | n.a. | n.a. | n.a. | n.a. | n.a. |
| 6467 | S | n.a. | n.a. | n.a. | n.a. | n.a. | n.a. | n.a. | n.a. | n.a. | n.a. | n.a. |
| 1138 | S | n.a. | n.a. | n.a. | n.a. | n.a. | n.a. | n.a. | n.a. | n.a. | n.a. | n.a. |
| 67 | S | n.a. | n.a. | n.a. | n.a. | n.a. | n.a. | n.a. | n.a. | n.a. | n.a. | n.a. |
| 5230 | S | n.a. | n.a. | n.a. | n.a. | n.a. | n.a. | n.a. | n.a. | n.a. | n.a. | n.a. |
| 5957 | S | n.a. | n.a. | n.a. | n.a. | n.a. | n.a. | n.a. | n.a. | n.a. | n.a. | n.a. |
| 6242 | S | n.a. | n.a. | n.a. | n.a. | n.a. | n.a. | n.a. | n.a. | n.a. | n.a. | n.a. |
| 5899 | S | n.a. | n.a. | n.a. | n.a. | n.a. | n.a. | n.a. | n.a. | n.a. | n.a. | n.a. |
| 222 | S | n.a. | n.a. | n.a. | n.a. | n.a. | n.a. | n.a. | n.a. | n.a. | n.a. | n.a. |
| 728 | S | n.a. | n.a. | n.a. | n.a. | n.a. | n.a. | n.a. | n.a. | n.a. | n.a. | n.a. |
| 1194 | S | n.a. | n.a. | n.a. | n.a. | n.a. | n.a. | n.a. | n.a. | n.a. | n.a. | n.a. |
| 5669 | S | n.a. | n.a. | n.a. | n.a. | n.a. | n.a. | n.a. | n.a. | n.a. | n.a. | n.a. |
| 283 | S | n.a. | n.a. | n.a. | n.a. | n.a. | n.a. | n.a. | n.a. | n.a. | n.a. | n.a. |
| 373 | S | n.a. | n.a. | n.a. | n.a. | n.a. | n.a. | n.a. | n.a. | n.a. | n.a. | n.a. |
| 5552 | S | n.a. | n.a. | n.a. | n.a. | n.a. | n.a. | n.a. | n.a. | n.a. | n.a. | n.a. |
| 5882 | S | n.a. | n.a. | n.a. | n.a. | n.a. | n.a. | n.a. | n.a. | n.a. | n.a. | n.a. |
| 6130 | S | n.a. | n.a. | n.a. | n.a. | n.a. | n.a. | n.a. | n.a. | n.a. | n.a. | n.a. |
| 5812 | S | n.a. | n.a. | n.a. | n.a. | n.a. | n.a. | n.a. | n.a. | n.a. | n.a. | n.a. |
| 303 | S | n.a. | n.a. | n.a. | n.a. | n.a. | n.a. | n.a. | n.a. | n.a. | n.a. | n.a. |
| 04DK | S | n.a. | n.a. | n.a. | n.a. | n.a. | n.a. | n.a. | n.a. | n.a. | n.a. | n.a. |
| 42 | S | n.a. | n.a. | n.a. | n.a. | n.a. | n.a. | n.a. | n.a. | n.a. | n.a. | n.a. |
| 1127 | S | n.a. | n.a. | n.a. | n.a. | n.a. | n.a. | n.a. | n.a. | n.a. | n.a. | n.a. |
| 543 | S | n.a. | n.a. | n.a. | n.a. | n.a. | n.a. | n.a. | n.a. | n.a. | n.a. | n.a. |
| 950 | S | n.a. | n.a. | n.a. | n.a. | n.a. | n.a. | n.a. | n.a. | n.a. | n.a. | n.a. |
| 5727 | S | n.a. | n.a. | n.a. | n.a. | n.a. | n.a. | n.a. | n.a. | n.a. | n.a. | n.a. |
| 146 | S | n.a. | n.a. | n.a. | n.a. | n.a. | n.a. | n.a. | n.a. | n.a. | n.a. | n.a. |
| 353 | S | n.a. | n.a. | n.a. | n.a. | n.a. | n.a. | n.a. | n.a. | n.a. | n.a. | n.a. |
| 6356 | S | n.a. | n.a. | n.a. | n.a. | n.a. | n.a. | n.a. | n.a. | n.a. | n.a. | n.a. |
| 877 | S | n.a. | n.a. | n.a. | n.a. | n.a. | n.a. | n.a. | n.a. | n.a. | n.a. | n.a. |
| 2OBK | S | n.a. | n.a. | n.a. | n.a. | n.a. | n.a. | n.a. | n.a. | n.a. | n.a. | n.a. |
| 195 | S | n.a. | n.a. | n.a. | n.a. | n.a. | n.a. | n.a. | n.a. | n.a. | n.a. | n.a. |
| 5894 | S | n.a. | n.a. | n.a. | n.a. | n.a. | n.a. | n.a. | n.a. | n.a. | n.a. | n.a. |
| 98 | S | n.a. | n.a. | n.a. | n.a. | n.a. | n.a. | n.a. | n.a. | n.a. | n.a. | n.a. |
| 5880 | S | n.a. | n.a. | n.a. | n.a. | n.a. | n.a. | n.a. | n.a. | n.a. | n.a. | n.a. |
| 888 | S | n.a. | n.a. | n.a. | n.a. | n.a. | n.a. | n.a. | n.a. | n.a. | n.a. | n.a. |
| 1148 | S | n.a. | n.a. | n.a. | n.a. | n.a. | n.a. | n.a. | n.a. | n.a. | n.a. | n.a. |
| 6444 | S | n.a. | n.a. | n.a. | n.a. | n.a. | n.a. | n.a. | n.a. | n.a. | n.a. | n.a. |
| 5727 | S | n.a. | n.a. | n.a. | n.a. | n.a. | n.a. | n.a. | n.a. | n.a. | n.a. | n.a. |
| 7009 | S | n.a. | n.a. | n.a. | n.a. | n.a. | n.a. | n.a. | n.a. | n.a. | n.a. | n.a. |
| 414 | S | n.a. | n.a. | n.a. | n.a. | n.a. | n.a. | n.a. | n.a. | n.a. | n.a. | n.a. |
| 5688 | S | n.a. | n.a. | n.a. | n.a. | n.a. | n.a. | n.a. | n.a. | n.a. | n.a. | n.a. |
| 5795 | S | n.a. | n.a. | n.a. | n.a. | n.a. | n.a. | n.a. | n.a. | n.a. | n.a. | n.a. |
| 5921 | S | n.a. | n.a. | n.a. | n.a. | n.a. | n.a. | n.a. | n.a. | n.a. | n.a. | n.a. |
| 5908 | S | n.a. | n.a. | n.a. | n.a. | n.a. | n.a. | n.a. | n.a. | n.a. | n.a. | n.a. |
| 879 | S | n.a. | n.a. | n.a. | n.a. | n.a. | n.a. | n.a. | n.a. | n.a. | n.a. | n.a. |
| 5506 | S | n.a. | n.a. | n.a. | n.a. | n.a. | n.a. | n.a. | n.a. | n.a. | n.a. | n.a. |
| 5654 | S | n.a. | n.a. | n.a. | n.a. | n.a. | n.a. | n.a. | n.a. | n.a. | n.a. | n.a. |
| 5601 | S | n.a. | n.a. | n.a. | n.a. | n.a. | n.a. | n.a. | n.a. | n.a. | n.a. | n.a. |
| 485 | S | n.a. | n.a. | n.a. | n.a. | n.a. | n.a. | n.a. | n.a. | n.a. | n.a. | n.a. |
| 382 | S | n.a. | n.a. | n.a. | n.a. | n.a. | n.a. | n.a. | n.a. | n.a. | n.a. | n.a. |
| 5922 | S | n.a. | n.a. | n.a. | n.a. | n.a. | n.a. | n.a. | n.a. | n.a. | n.a. | n.a. |
| 5934 | S | n.a. | n.a. | n.a. | n.a. | n.a. | n.a. | n.a. | n.a. | n.a. | n.a. | n.a. |
| 5877 | S | n.a. | n.a. | n.a. | n.a. | n.a. | n.a. | n.a. | n.a. | n.a. | n.a. | n.a. |
| 6443 | S | n.a. | n.a. | n.a. | n.a. | n.a. | n.a. | n.a. | n.a. | n.a. | n.a. | n.a. |
| 3OBK | S | n.a. | n.a. | n.a. | n.a. | n.a. | n.a. | n.a. | n.a. | n.a. | n.a. | n.a. |
| 5641 | S | n.a. | n.a. | n.a. | n.a. | n.a. | n.a. | n.a. | n.a. | n.a. | n.a. | n.a. |
| 289 | S | n.a. | n.a. | n.a. | n.a. | n.a. | n.a. | n.a. | n.a. | n.a. | n.a. | n.a. |
| 380 | S | n.a. | n.a. | n.a. | n.a. | n.a. | n.a. | n.a. | n.a. | n.a. | n.a. | n.a. |
| 847 | S | n.a. | n.a. | n.a. | n.a. | n.a. | n.a. | n.a. | n.a. | n.a. | n.a. | n.a. |
| 5533 | S | n.a. | n.a. | n.a. | n.a. | n.a. | n.a. | n.a. | n.a. | n.a. | n.a. | n.a. |
| 369EING | S | n.a. | n.a. | n.a. | n.a. | n.a. | n.a. | n.a. | n.a. | n.a. | n.a. | n.a. |
| 5962 | S | n.a. | n.a. | n.a. | n.a. | n.a. | n.a. | n.a. | n.a. | n.a. | n.a. | n.a. |
| 6349 | S | n.a. | n.a. | n.a. | n.a. | n.a. | n.a. | n.a. | n.a. | n.a. | n.a. | n.a. |
| 5924 | S | n.a. | n.a. | n.a. | n.a. | n.a. | n.a. | n.a. | n.a. | n.a. | n.a. | n.a. |
| 5505 | S | n.a. | n.a. | n.a. | n.a. | n.a. | n.a. | n.a. | n.a. | n.a. | n.a. | n.a. |
| 777 | S | n.a. | n.a. | n.a. | n.a. | n.a. | n.a. | n.a. | n.a. | n.a. | n.a. | n.a. |
| 6011 | S | n.a. | n.a. | n.a. | n.a. | n.a. | n.a. | n.a. | n.a. | n.a. | n.a. | n.a. |
| 323 | S | n.a. | n.a. | n.a. | n.a. | n.a. | n.a. | n.a. | n.a. | n.a. | n.a. | n.a. |
| 923 | S | n.a. | n.a. | n.a. | n.a. | n.a. | n.a. | n.a. | n.a. | n.a. | n.a. | n.a. |
| 5584 | S | n.a. | n.a. | n.a. | n.a. | n.a. | n.a. | n.a. | n.a. | n.a. | n.a. | n.a. |
| 74 | S | n.a. | n.a. | n.a. | n.a. | n.a. | n.a. | n.a. | n.a. | n.a. | n.a. | n.a. |
| 5494 | S | n.a. | n.a. | n.a. | n.a. | n.a. | n.a. | n.a. | n.a. | n.a. | n.a. | n.a. |
| 5517 | S | n.a. | n.a. | n.a. | n.a. | n.a. | n.a. | n.a. | n.a. | n.a. | n.a. | n.a. |
| 5916 | S | n.a. | n.a. | n.a. | n.a. | n.a. | n.a. | n.a. | n.a. | n.a. | n.a. | n.a. |
| 82 | S | n.a. | n.a. | n.a. | n.a. | n.a. | n.a. | n.a. | n.a. | n.a. | n.a. | n.a. |
| 5478 | S | n.a. | n.a. | n.a. | n.a. | n.a. | n.a. | n.a. | n.a. | n.a. | n.a. | n.a. |
| 764 | S | n.a. | n.a. | n.a. | n.a. | n.a. | n.a. | n.a. | n.a. | n.a. | n.a. | n.a. |
| 5725 | S | n.a. | n.a. | n.a. | n.a. | n.a. | n.a. | n.a. | n.a. | n.a. | n.a. | n.a. |
| 606 | S | n.a. | n.a. | n.a. | n.a. | n.a. | n.a. | n.a. | n.a. | n.a. | n.a. | n.a. |
| 954 | S | n.a. | n.a. | n.a. | n.a. | n.a. | n.a. | n.a. | n.a. | n.a. | n.a. | n.a. |
| 321 | S | n.a. | n.a. | n.a. | n.a. | n.a. | n.a. | n.a. | n.a. | n.a. | n.a. | n.a. |
| 378 | S | n.a. | n.a. | n.a. | n.a. | n.a. | n.a. | n.a. | n.a. | n.a. | n.a. | n.a. |
| 5960 | S | n.a. | n.a. | n.a. | n.a. | n.a. | n.a. | n.a. | n.a. | n.a. | n.a. | n.a. |
| 6500 | S | n.a. | n.a. | n.a. | n.a. | n.a. | n.a. | n.a. | n.a. | n.a. | n.a. | n.a. |
| 5459 | S | n.a. | n.a. | n.a. | n.a. | n.a. | n.a. | n.a. | n.a. | n.a. | n.a. | n.a. |
| 6817 | S | n.a. | n.a. | n.a. | n.a. | n.a. | n.a. | n.a. | n.a. | n.a. | n.a. | n.a. |
| 5835 | S | n.a. | n.a. | n.a. | n.a. | n.a. | n.a. | n.a. | n.a. | n.a. | n.a. | n.a. |
| 5748 | S | n.a. | n.a. | n.a. | n.a. | n.a. | n.a. | n.a. | n.a. | n.a. | n.a. | n.a. |
| 1164 | S | n.a. | n.a. | n.a. | n.a. | n.a. | n.a. | n.a. | n.a. | n.a. | n.a. | n.a. |
| 5485 | S | n.a. | n.a. | n.a. | n.a. | n.a. | n.a. | n.a. | n.a. | n.a. | n.a. | n.a. |
| 6281 | S | n.a. | n.a. | n.a. | n.a. | n.a. | n.a. | n.a. | n.a. | n.a. | n.a. | n.a. |
| 110 | S | n.a. | n.a. | n.a. | n.a. | n.a. | n.a. | n.a. | n.a. | n.a. | n.a. | n.a. |
| 1244 | S | n.a. | n.a. | n.a. | n.a. | n.a. | n.a. | n.a. | n.a. | n.a. | n.a. | n.a. |
| 79 | S | n.a. | n.a. | n.a. | n.a. | n.a. | n.a. | n.a. | n.a. | n.a. | n.a. | n.a. |
| 411 | S | n.a. | n.a. | n.a. | n.a. | n.a. | n.a. | n.a. | n.a. | n.a. | n.a. | n.a. |
| 5685 | S | n.a. | n.a. | n.a. | n.a. | n.a. | n.a. | n.a. | n.a. | n.a. | n.a. | n.a. |
| 925ED | S | n.a. | n.a. | n.a. | n.a. | n.a. | n.a. | n.a. | n.a. | n.a. | n.a. | n.a. |
| 6481 | S | n.a. | n.a. | n.a. | n.a. | n.a. | n.a. | n.a. | n.a. | n.a. | n.a. | n.a. |
| 5541 | S | n.a. | n.a. | n.a. | n.a. | n.a. | n.a. | n.a. | n.a. | n.a. | n.a. | n.a. |
| 5712 | S | n.a. | n.a. | n.a. | n.a. | n.a. | n.a. | n.a. | n.a. | n.a. | n.a. | n.a. |
| 309 | S | n.a. | n.a. | n.a. | n.a. | n.a. | n.a. | n.a. | n.a. | n.a. | n.a. | n.a. |
| 280 | S | n.a. | n.a. | n.a. | n.a. | n.a. | n.a. | n.a. | n.a. | n.a. | n.a. | n.a. |

**Legend**: RIF: rifampicin; INH: Isoniazid; PZA: pyrazinamide; FQs: fluoroquinolones; SL-INJ: second line-Injectables; KAN: kanamycin; CAPREO: capreomycin; FQs: fluoroquinolones; fs = frameshift; Del = deletion

**Note 1**: only drug resistance conferring mutation were reported.
